# The Immunomodulatory Properties of the Human Amnion-Derived Mesenchymal Stromal/Stem Cells Are Induced by INF-γ Produced by Activated Lymphomonocytes and Are Mediated by Cell-To-Cell Contact and Soluble Factors

**DOI:** 10.3389/fimmu.2020.00054

**Published:** 2020-02-12

**Authors:** Matteo Bulati, Vitale Miceli, Alessia Gallo, Giandomenico Amico, Claudia Carcione, Mariangela Pampalone, Pier Giulio Conaldi

**Affiliations:** ^1^Research Department, Mediterranean Institute for Transplantation and Advanced Specialized Therapies (IRCCS ISMETT), Palermo, Italy; ^2^Ri.MED Foundation, Palermo, Italy

**Keywords:** human amnion-derived mesenchymal stem cells, immunomodulation, interferon-γ, primed-hAMSCs, regenerative medicine, exosomes, M2-like monocytes, PDL-1

## Abstract

Human mesenchymal stromal/stem cells (MSCs), being immunoprivileged and having immunomodulatory ability, represent a promising tool to be applied in the field of regenerative medicine. Based on numerous *in vitro* evidences, the immunological effects of MSCs on immune cells could depend on different mechanisms as cell-to-cell contact and paracrine signals. Furthermore, recent studies have shown that the immunomodulatory activity of MSCs is initiated by activated immune cells; thus, their interaction represents a potential homeostatic mechanism by which MSCs regulate the immune response. MSCs also release exosomes able to give different effects, in a paracrine manner, by influencing inflammatory processes. In this study, we aimed to establish the potential role of human amnion-derived MSCs (hAMSCs), in immunomodulation. We found that the immunosuppressive properties of hAMSCs are not constitutive, but require “supportive signals” capable of promoting these properties. Indeed, we observed that hAMSCs alone are not able to produce an adequate amount of soluble immunomodulatory factors. Here, we studied, in depth, the strong immunomodulatory licensing signal deriving from the direct interaction between hAMSCs and stimulated peripheral blood mononuclear cells. We found that the immunomodulatory effect of hAMSCs also depends on cell-to-cell contact through the contribution of the PDL-1/PD-1 axis. We then investigated the IFN-γ priming of hAMSCs (γ-hAMSCs), which induce the increase of PDL-1 expression, high production of IDO, and upregulation of different immunomodulatory exosome-derived miRNAs. Our miRNA–target network analysis revealed that nine of the deregulated miRNAs are involved in the regulation of key proteins that control both T cell activation/anergy and monocyte differentiation pathways. Finally, we observed that γ-hAMSCs induce in monocytes both M2-like phenotype and the increase of IL-10 production. The extensive implications of MSCs in modulating different aspects of the immune system make these cells attractive candidates to be employed in therapeutic application in immune-based diseases. For these reasons, we aimed, with this study, to shed light on the potential of hAMSCs, and how they could become a useful tool for treating different inflammatory diseases, including end-stage pathologies or adverse effects in transplanted patients.

## Introduction

Mesenchymal stromal/stem cells (MSCs) can be defined as a heterogeneous population of adult fibroblast-like multipotent cells, which can be isolated from different biological sources. These cells are expanded *ex vivo* in culture, and are considered an important component for physiological remodeling and tissue repair (1–3). MSCs reside in all connective tissues, but can also be isolated from fetal or adult somatic tissues, such as amniotic membrane (4), umbilical cord (5), bone marrow (6), adipose tissue (7), fetal liver (8), fetal lung (9), and tooth pulp (10). Because of their different tissue origins, there is still no standard procedure for the univocal identification of these cells, despite a consensus for the three minimum criteria to identify MSCs proposed by the International Committee for Cell Therapy (ISCT) (11). First, these cells must have plastic adherent fibroblast-like growth properties when they are maintained in standard culture conditions. Second, MSCs must bear on their surface a set of specific antigens, such as CD73, CD105, CD90, CD44, CD13, and CD71 with the simultaneous lack of the typical hematopoietic markers CD45, CD34, CD14, CD19, CD79a, and HLA-DR, and of co-stimulatory molecules such as CD40, CD80, and CD86. Finally, using appropriate culture media, MSCs can be induced to differentiate *in vitro* into adipocyte-, chondroblast-, or osteoblast-like cells (12).

The scientific and clinical interest in MSCs derives from their potential therapeutic values given by their peculiar biological properties, such as high proliferative capacity, ability to differentiate into many somatic cell lineages, and ability to migrate and home to inflamed or injured tissues, and because of their powerful capacity to modulate the immune system response (3, 13). MSCs, with their regenerative ability and immunomodulatory function, have been used for inflammatory and degenerative disease treatments (3).

The peculiar immunomodulatory properties of MSCs, together with the lack or low expression of major histocompatibility complex II antigens (HLA-DR), and co-stimulatory molecules (CD80, CD86) on their surface, render these cells able to induce suppression of the host immune response when used in allogeneic settings (1), giving to these cells an immune privilege status. MSCs can affect different pathways of the immune system response in a paracrine way, producing soluble factors, and through cell-to-cell contacts (1).

At present, the principal molecular and cellular mechanisms of the MSCs' immunosuppressive effect are still under investigation, together with the effects of allogeneic immune cells on MSC characteristics, which have not been adequately studied. To evaluate the immunomodulatory activity of MSCs, it is necessary to take into account the local microenvironment in which these cells exert their functions. First, because MSCs are equipped with different toll-like receptors (TLR) (14), these cells can be exposed to TLR ligands or “dangerous signals,” such as heat shock protein 70 (HSP-70), hyaluronic acid fragments, fibronectin extra domain A, and oxidized LDL, produced upon injury at the sites of inflammation. These events can lead to the activation of MSC TLRs and, as a consequence, to a different response mode (15), even if the data in the literature on this argument are discrepant (16). Furthermore, the immunosuppressive behavior of MSCs can also be influenced by the pro-inflammatory cytokines produced by activated immune cells. To this purpose, it has been demonstrated that the activation of MSCs by certain pro-inflammatory cytokines (IFN-γ, TNF-α, IL-1α, and IL-1β), produced by stimulated immune cells, is necessary for the manifestation of their immunosuppressive properties (17–20).

Among MSCs, the ones extracted from placenta show several advantages. As placenta is spontaneously expulsed *post-partum*, the use of invasive procedures, as in the case of adult stem cells deriving from other sources, is not necessary. Different from the use of embryonic stem cells, there are no ethical concerns in its use, because placenta is considered a medical waste (21). Moreover, there is also an advantage in terms of immunomodulation, as placenta is the immunoregulatory organ at the maternal–fetal interface (22). Depending on the layer of origin, placenta contains different populations of MSCs, such as amnion MSCs (AMSCs), chorion MSCs (CMSCs), chorionic villi MSCs (CV-MSCs), and decidua MSCs (DMSCs) (4, 23–25).

In this research, we studied the immune-like behavior of human amnion-derived MSCs (hAMSCs) and the cross-talk between these cells and the immune system cells. We first evaluated the basal production by hAMSCs of some paracrine factors important for the immunosuppressive response. Subsequently, after evaluating the presence of some TLRs expressed by hAMSCs, we assessed whether and how pre-activating with TLR-agonists, their immunosuppressive behavior could be changed. Then, with the aim of better understanding the immunomodulatory mechanisms of hAMSCs in an allogeneic setting, we further investigated the ability of hAMSCs to inhibit the *in vitro* proliferation of peripheral blood mononuclear cells (PBMCs), or the effect of hAMSCs on specific populations of the immune system. We highlight how the hAMSC function is strongly influenced by the immune system cell activation, which constitutes a “negative feedback mechanism” for immune response itself. Activated immune cells produce soluble molecules such as IFN-γ, which enhance the immunosuppressive capacity of hAMSCs (19, 26). Indeed, in hAMSC/PBMC co-culture, or in hAMSCs primed with IFN-γ, we found high concentrations of soluble factors, such as indolamine 2,3-dioxygenase (IDO), prostaglandin E_2_ (PGE_2_), and interleukin-10 (IL-10). In our previous report (27), we showed an inhibitory capacity of hAMSC-derived exosomes (EXO) on PBMC proliferation. We also investigated the molecular mechanisms potentially involved in EXO immune suppression action.

Other than by soluble factors, MSC-mediated immunosuppression involves cell contact-dependent mechanisms through different surface proteins (26). Among these proteins, programmed cell death ligand-1 (PDL-1/CD274), which is constitutively expressed on MSCs, seems to be the natural candidate involved in the induction of the cell-to-cell contact immunomodulation by hAMSCs. When the PDL-1 binds its ligand, programmed cell death-1 (PD-1), which is expressed on activated T and B cells, activates an immunosuppressive response (28–31). In this study, we show the involvement of the (PDL-1)/(PD-1) pathway axis in the suppression of the immune response by hAMSCs.

Moreover, we also found a remodeling of the regulatory immune system cells toward an anti-inflammatory phenotype, with an increase of M2-like CD14^+^/CD206^+^ monocytes. In particular, these cells, which are induced in co-culture with hAMSCs pre-activated with IFN-γ, themselves acquire the ability to suppress immune system activation.

The results on this study could provide a useful method for obtaining functionally qualified hAMSCs that can appropriately modulate the immune response. In this way, hAMSCs could become a useful tool for treating end-stage organ pathology in which immune-related disorders are involved, or in the pre-treatment of patients who are candidates for transplantation to attenuate the inflammatory status before the transplant.

## Materials and Methods

### Isolation and Culture of Human Amnion Mesenchymal Stem Cells

To obtain hAMSCs, written informed consent was obtained from each donor, and the procedure was approved by IRCCS-ISMETT's Institutional Research Review Board (IRRB). hAMSCs were isolated from amnion of human term placenta (38–40 weeks of gestation) of healthy donors within 6 h of birth, using a previously described protocol (32). Briefly, the amnion was manually separated from the chorion and washed several times in phosphate-buffered saline (PBS). It was then cut into small pieces of 3 × 3 cm^2^, and each fragment was decontaminated with a brief incubation in PBS with 2.5% Esojod (Esoform, Italy) for 3 min in PBS containing 500 U/ml penicillin, 500 mg/ml streptomycin, 12.5 mg/ml amphotericin B, 1.87 mg/ml cefamezin (Pfizer, Italy), and 5 min in PBS containing 100 U/ml penicillin and 100 mg/ml streptomycin. Decontaminated fragments were incubated for 9 min at 37°C in HBSS (Lonza, CH) containing 2.5 U/ml dispase (Corning, NY, USA). The fragments were then incubated for 5 min at room temperature in complete RPMI 1640 medium (Sigma-Aldrich) supplemented with 10% fetal bovine serum (FBS) (Sigma-Aldrich), and subsequently digested with 0.94 mg/ml collagenase A (Roche, Germany) and 20 mg/ml DNase (Roche, Germany) for 2.5 h at 37°C. The digest was subsequently filtered with both 100 and 70 μm cell strainer (BD Falcon, USA), pelleted by centrifugation at 150–300 g for 10 min, and re-suspended in a complete RPMI 1640 medium (Sigma-Aldrich) supplemented with 10% fetal bovine serum (FBS) (Sigma-Aldrich) for evaluation of cell number, viability, and phenotype characterization. hAMSCs were cultured in plastic dishes at ~10^5^ cells/cm^2^ using Chang Medium (Irvine Scientific) supplemented with 1% glutamine and 1% antibiotics at 37°C and 5% CO_2_. Cell growth was monitored and attached cells reached confluence 10 days after plating (passage 0). Cells were then trypsinized using a 0.05% trypsin−0.5 mM EDTA solution (Euroclone), and expanded into a T-175 flask for four passages. The culture medium was changed every 3–4 days, and the cells were split approximately 1:4 at each subsequent passage. For our experiment we used cells from passage 4 to passage 5.

### Preparation of Immune Cells

A total of 10 healthy volunteers were enrolled in this study. All subjects give written informed consent in accordance with the Declaration of Helsinki. PBMCs were isolated from venous blood by density gradient centrifugation on Lympholyte Cell Separation Media (Cedarlane Laboratories Limited, Ontario, Canada). PBMCs were adjusted to 1 × 10^6^/ml in RPMI 1640 medium (Sigma-Aldrich) supplemented with 10% heat-inactivated fetal bovine serum (Sigma-Aldrich), 1% penicillin/streptomycin (Sigma-Aldrich), 10 mM HEPES (Euroclone), and 1 mM L-glutamine (Lonza).

CD14^+^ monocytes or CD3^+^ T lymphocytes were separated from PBMCs by immunomagnetic sorting using anti-CD14 (MACS CD14 Microbeads; Miltenyi Biotec, Auburn, CA, USA) or anti-CD3 (MACS CD3 Microbeads; Miltenyi Biotec, Auburn, CA, USA) magnetic microbeads. The cells obtained from immunomagnetic sorting were >98% CD14^+^ monocytes or CD3^+^ lymphocytes, as determined by flow cytometry analysis.

### Cell Treatment and Culture

For TLR3 or TLR4 triggering, cultured hAMSCs were activated, with 20 μg/ml of poly (I:C) (Miltenyi Biotec) or 2 μg/ml of LPS (Sigma-Aldrich), for 24 or 48 h. In some experiments, hAMSCs were cultured for 48 h in the presence of 200 IU/ml of IFN-γ (Human IFN-g1b premium grade, Miltenyi Biotec).

PBMCs or magnetically sorted CD3^+^ T lymphocytes were activated with anti-human CD3 (0.5 μg/ml, CD3 pure, human-functional grade, Miltenyi Biotec) and anti-human CD28 (0.5 μg/ml, CD28 pure, human-functional grade, Miltenyi Biotec) for 5 days. Only PBMCs were further stimulated with anti-human CD40 (0.5 μg/ml, CD40 pure, human-functional grade, Miltenyi Biotec) and 2 μg/ml anti-BCR [affiniPure F(ab)′2 Fragment Goat Anti-Human IgA + IgG + IgM, Jackson ImmunoResearch Laboratories, Inc., Philadelphia] to also induce B cells activation.

For culture assays, hAMSCs were always seeded at a density of 1 × 10^5^ cells in 24-well plate tissue culture treated with or without (6.55 mm, 0.4 μm polyester membrane) Transwell insert (Costar) in RPMI 1640 medium supplemented with 10% heat-inactivated fetal bovine serum, 1% penicillin/streptomycin, 10 mM HEPES, and 1 mM L-glutamine. In some experiments, hAMSCs were co-cultured with 5 × 10^5^ activated or non-activated PBMC or immune-magnetically sorted CD14^+^ or CD3^+^ cells.

### Isolation and Characterization of Exosomes (EXO)

**Figure 1:**
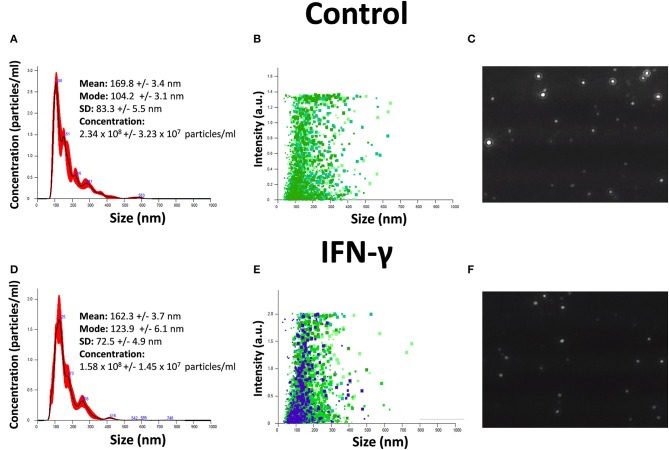
Characterization of EXO secreted by hAMSCs. **(A)** Size and concentration of EXO isolated from hAMSCs. **(B)** Size and intensity of EXO isolated from hAMSCs. **(C)** Representative images of EXO isolated from hAMSCs. **(D)** Size and concentration of EXO isolated from hAMSCs treated with IFNγ. **(E)** Size and intensity of EXO isolated from hAMSCs treated with IFNγ. **(F)** Representative images of EXO isolated from hAMSCs treated with IFNγ.

EXO were isolated from hAMSC cultures at 90% confluence. The serum-free conditioned medium used was collected after 2 days of culture and centrifuged at 300 × *g* for 10 min to remove the debris. The medium was then centrifuged for 20 min at 17,000 × *g* and ultracentrifuged at 150,000 × *g* for 90 min at 4°C to pellet the EXO. Both size distribution and concentration of EXO were determined by NTA in a NanoSight NS3000 (Malvern Instruments Ltd, Malvern, UK). Samples were diluted 80 times with PBS to reach optimal concentration for instrument linearity, and the data were analyzed with NTA software version 3.1 (Figure 1). Readings were taken on triplicates of 60 s at 25 frames per second, at a camera level set to 16, and with manual monitoring of temperature. The pellets containing EXO were then processed to extract RNA.

### Labeling of PBMCs or Magnetically Sorted CD3^+^ T Lymphocytes

PBMCs, or isolated CD3^+^ T lymphocytes, were labeled with carboxy-fluorescein succinimidyl ester (CFSE) (Cell-Trace CFSE Proliferation Kit, Molecular Probes, Invitrogen) in order to monitor their proliferation by flow cytometry. Briefly, 6 × 10^6^ cells (PBMCs or magnetically sorted CD3^+^ lymphocytes) were resuspended in 2 ml of PBS, and 1 μl of 5 mM CFSE was added (final concentration, 2.5 μM), mixed well, and incubated for 10 min in darkness at 37°C in a humidified CO_2_ atmosphere (5%). The reaction was then stopped by adding 10 ml of RPMI 1640 medium with 10% FBS, followed by centrifugation. This wash step was repeated two times, and cells were finally resuspended in RPMI 1640 medium with 10% FBS until their use for the cell proliferation. CFSE-labeled cells were seeded onto flat-bottomed 24-well plates in either the presence or absence of hAMSCs. In some experiments, PBMCs and hAMSCs were separated by a 6.55-mm (0.4 μm polyester membrane) Transwell insert (Costar). Proliferation of PBMCs, or CD3^+^ T lymphocytes, was determined by measuring the reduction of fluorescence intensity at day 5 after stimulation with anti-human CD3 and anti-human CD28, for both PBMCs and CD3 lymphocytes, while for only PBMCs, anti-human CD40 and anti-BCR were added. Basal fluorescence was determined after an overnight CFSE staining. To determine the effect of hAMSCs on the inhibition of PBMC or CD3^+^ T lymphocyte proliferation, data were normalized considering the stimulated condition of single PBMCs, or CD3^+^ lymphocytes culture (without hAMSCs) as 100% of proliferation. After flow cytometer acquisition, data were analyzed with ModFit LT version 3.0 software (Verity Software House).

### Flow Cytometry Analysis

hAMSC, total PBMCs, and magnetically sorted CD3^+^ T lymphocytes or CD14^+^ monocytes were stained with different combinations of the following monoclonal antibodies: anti-human CD45_PerCP−Cyanine5.5_, anti-human CD274(PDL-1)_PerCP−eFluor710_ (eBioscience, Thermofisher), anti-human CD40_PE_, anti-human CD80_FITC_ or CD80_PECy5_, anti-human CD86_PE_ (Pharmingen, BD Bioscience, Mountain View, CA, USA), anti-human HLA-DR_FITC_, anti-human CD3_PerCP_, anti-human CD14_APCVio770_, anti-human CD73_APC_, anti-human CD206_APC_, anti-human CD273(PDL-2)_PE−Vio770_, anti-human CD279(PD-1)_VioBrightFITC_, anti-human CD283(TLR3)_PE_, and anti-human CD284(TLR4)_APC_ (Miltenyi Biotec).

All measurements were made with a FACSCanto II flow cytometer (Becton Dickinson, San Jose, CA, USA) with the same instrument setting. At least 10^4^ cells were analyzed using FACSDiva 8.0.1 (Becton Dickinson, San Jose, CA, USA) software.

### RNA Interference Assay

The transfected cells were seeded at 0.5–2 × 10^5^ cells/well into 24-well dishes and cultured overnight until they reached 70% confluence. Transfections were performed using Lipofectamine^®^ RNAiMAX Reagent (Thermo Fischer Scientific, MA, USA) following the manufacturer's instructions. Ten nanomoles of RNAi (Silencer^®^ Select TLR3 ID#s235, Silencer^®^ Select TLR4 ID#s14196, Silencer^®^ Select TLR9 ID#s28872, Silencer^®^ Select CD274 ID#s26549, and scrambled siRNA # 4390843) (Thermo Fischer Scientific, MA, USA) was used for transfection into cells. Transfected cells were incubated for 48 h, and mRNA levels of TLR3, TLR4, TLR9, and CD274 transcripts were then measured.

### miRNAs Transfection of Monocytes

Transfection assays were performed using Hiperfect Transfection Reagent according to the manufacturer's instructions (Qiagen, Hilden, Germany). 2 × 10^5^ purified monocytes were transfected for 48 h with 50 nM of miRCURY LNA™ miRNA-miR-130b-3p, -miR-26b-5p, -miR-125b-5p, -miR-203a-3p, -miR-23a-3p, and -miR-223-3p (Qiagen, Hilden, Germany), and their corresponding miRCURY LNA™ miRNA Inhibitor (Qiagen, Hilden, Germany), as well as scrambled sequences (Qiagen, Hilden, Germany). Briefly, 3 μl of Hiperfect Transfection Reagent was added to 100 μl of serum-free RPMI medium containing mimics alone or different combinations of them, with or without their corresponding inhibitor, scrumble, and negative control at a final concentration of 50 nM.

### Real-Time PCR

Total RNA was extracted with the RNeasy Mini Kit and treated with DNAse (Qiagen, Hilden, Germany). Subsequently, 100 ng of RNA was reverse-transcribed with the high capacity RNA-to-cDNA kit protocol (Thermo Fisher Scientific, MA, USA) in order to produce single-stranded cDNA.

We performed real-time PCR using cDNA as the template in a 20-μl reaction mixture containing SYBR Select Master Mix (Life Technologies, USA) and a specific primer pair for the following genes: *IP10* (F-CAAGCCAATTTTGTCCACGT; R-GTAGGGAAGTGATGGGAGAG; Seq. ID: NM_001565.3), *IDO* (F-GCCAGCTTCGAGAAAGAGTTG; R-TGACTTGTGGTCTGTGAGATGA; Seq. ID: NM_002164.5), *IL10* (F-GTGATGCCCCAAGCTGAGA; R-CACGCCTTGCTCTTGTTTT; Seq. ID: NM_000572.2), *IL6* (F-TGTGAAAGCAGCAAAGAGGC; R-TGGGTCAGGGGTGGTTATT; Seq. ID: NM_000600.4), *COX-2* (F-TGAGTGTGGGATTTGACCAG; R-TGTGTTTGGAGTGGGTTTCA; Seq. ID: NM_000963.3), *TNF*-α (F-GGCGTGGAGCTGAGAGATAAC; R-GGTGTGGGTGAGGAGCACAT; Seq. ID: NM_000594.3), and *GAPDH* (F-TCAAGAAGGTGGTGAAGCAGG; R-ACCAGGAAATGAGCTTGACAAA; Seq. ID: NM_002046.6). We also used the TaqMan gene assay (Life Technologies, USA) for analysis of TLR3 (#Hs00152933_m1), TLR4 (#Hs00152939_m1), TLR9 (#Hs00152973_m1), CD274 (Hs00204257_m1), and GAPDH (#Hs99999905_m1).

Expression of mRNA was quantified by PCR using StepOnePlus Real-Time PCR System (Applied Biosystems, Thermo Fisher Scientific, MA, USA). GAPDH was used as a reference gene for the relative quantification, assessed by 2^−ΔΔCT^ calculation for each mRNA. The analysis of EXO miRNA profiling was done with TaqMan Array Human MicroRNA panels A and B (Life Technologies, Thermo Fisher Scientific) to analyze 754 human miRNAs. Total RNA was extracted from the EXO pellet using the QIAzol Lysis Reagent (Qiagen), as previously described (33). The purity of isolated RNA was determined by OD260/280 using a NanoDrop ND-1000 Spectrophotometer (Thermo Scientific, Worcester, MA). Reverse transcription and pre-amplification were done following the manufacturer's instructions (Life Technologies, Thermo Fisher Scientific). QRT-PCR was performed with the Applied Biosystems 7900 HT Real-Time PCR system. For each miRNA, the expression level was determined with the equation 2^−ΔΔCT^. The U6 was used as housekeeping gene. Furthermore, hierarchical cluster analysis of miRNA expression was used to group treatments with a similar expression pattern. The *Z* scores were used for clustering miRNAs. The expression measurements of each gene were converted to *Z* scores by subtracting the mean value of the given gene, and dividing by the corresponding standard deviation (SD), thus bringing the measurements of every gene to a common scale. miRNA expression data were grouped using a hierarchical clustering algorithm in the Cluster 3.0 program. A heat map was generated using the Java TreeView program.

### Western Blot

Cells were treated with RIPA buffer and protease inhibitors (Protease Inhibitor Cocktail, Merck, Germany), and protein concentration was determined by Qubit^®^ 3.0 fluorometer (Life Technologies). Proteins were separated on 4–12% SDS-PAGE gel (Thermo Fisher Scientific, Rockford, USA) and transferred to PVDF membranes. Membranes were blotted overnight with antibodies against IKK (polyclonal antibody, GeneTex, Inc. North America) and IRF-1 (polyclonal antibody, GeneTex, Inc. North America). The membranes were washed and incubated with anti-rabbit HRP-conjugated secondary antibodies, purchased from Cell Signaling Technology. The signal was captured using a ChemiDoc XRS (BioRAD, CA, USA).

### Profiling of Paracrine Factors

The levels of different paracrine factors (*IDO; IL-10; IFN*-γ*; TNF*-α*; IP-10; MIG; MIP-1*α*; MIP-1*β) involved in inflammation were determined using magnetic beads technology from Luminex™ with the ProcartaPlex Human Cytokine Chemokine Growth Factor (Affymetrix, Vienna, Austria), according to the manufacturer's instructions. In addition, the levels of *PGE*_2_ were determined using the prostaglandin E2 parameter assay kit (R&D Systems, USA), following the manufacturer's instructions. Concentration of each factor was calculated from standards curves.

### Target Gene Prediction

The miRNET database (https://www.mirnet.ca/faces/home.xhtml) (34) is a comprehensive atlas of miRNA–target interactions that can integrate the information resulting from 11 existing miRNA–target prediction programs (TarBase, miRTarBase, miRecords, miRanda, miR2Disease, HMDD, PhenomiR, SM2miR, PharmacomiR, EpimiR, and starBase). In this study, miRNET was employed to predict the target genes of the significantly upregulated miRNAs. To investigate the functional implications of miRNA deregulations in γ-hAMSCs, we created a protein–protein interaction network of molecules targeted by at least two of our deregulated miRNAs, which are directed toward at least three genes, with pathway annotations in miRNET.

### Statistics

All data were analyzed from at least three independent experiments and expressed as mean ± SD. Data from different groups were compared using computerized statistical software with the ANOVA test. When ANOVA revealed a *p* < 0.05, the data were further analyzed with Dunnett's *t*-test. Differences were considered statistically significant at *p* < 0.05.

## Results

### Resting or TLRs-Primed hAMSCs Produce Low Levels of Immunomodulatory Paracrine Factors

**Figure 2:**
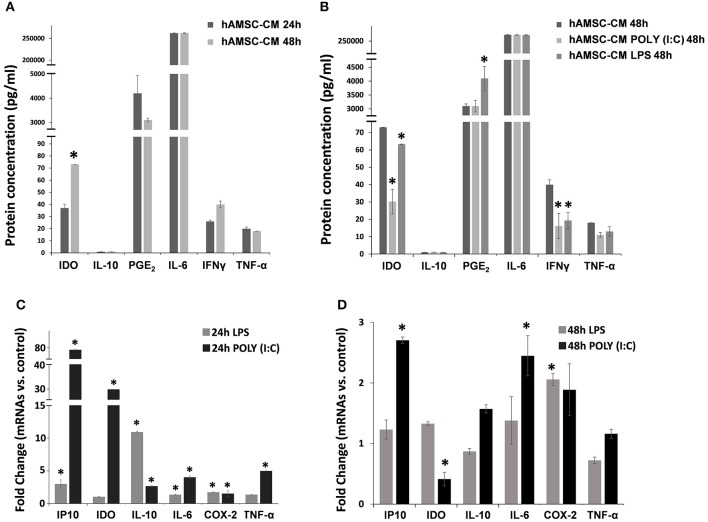
Expression analysis of immunomodulatory factors. Both protein **(A,B)** and gene **(C,D)** expression were assayed after 24 or 48 h of cultures in CM and cells, respectively. **(A)** Protein expression of immunomodulator factor in hAMSCs cultured for both 24 and 48 h. **(B)** Protein expression of immunomodulator factor in hAMSCs cultured for 48 h and treated with poly (I:C) or LPS. **(C)** Gene expression of immunomodulator factor in hAMSCs cultured for 24 h and treated with poly (I:C) or LPS. **(D)** Gene expression of immunomodulator factor in hAMSCs cultured for 48 h and treated with poly (I:C) or LPS. Transcript levels were normalized to those of GAPDH, and expressed as fold change vs. untreated cells (control). Data are means ± SD. ^*^p < 0.05 vs. control.

To test their intrinsic immunosuppressive activity, hAMSCs were cultured in complete medium, and supernatants were collected after 24 and 48 h of culture. As shown in Figure 2, hAMSCs produce high levels of IL-6, at both time points, without any significant differences. Other paracrine factors, such as IL-10, PGE_2_, IFN-γ, and TNF-α, are produced at very low levels, without any significant difference between the two time points. On the contrary, a significant increase of IDO after 48 h of incubation was observed (Figure 2A). It is known that MSCs are equipped with several TLRs that can modify their behavior in terms of differentiation, migration ability, immunomodulatory function, and therapeutic potency (35). For this reason, we first evaluated the expression of three different TLRs on hAMSCs: TLR3, TLR4, and TLR9. We found the mRNA expression of TLR3 and TLR4, but not of TLR9, which was also confirmed by the presence of the surface (TLR4) and intracellular (TLR3) receptor expression on hAMSCs by flow cytometry (not shown). To study the effect of TLR activation on hAMSCs, we treated cells with either poly (I:C) or LPS for TLR3 or TLR4 triggering, respectively. First, we assessed whether, after the TLR engagement, hAMSCs retain their immune privilege properties. We found that, even after TRL triggering, hAMSCs do not express either HLA-DR or co-stimulatory molecules such as CD80, CD86, and CD40 (not shown), demonstrating that hAMSCs still preserve their immune privilege status. To evaluate the effect of TLR triggering on hAMSC soluble factor production, cells were cultured on complete medium with or without LPS or poly (I:C) pre-treatment. After 48 h of culture, supernatants were collected and analyzed to evaluate the production of the same paracrine factors evaluated in hAMSC resting condition. As shown in Figure 2B, no changes were observed either for IL-6 or for IL-10, while both LPS and poly (I:C) induced significantly reduced production of IFN-γ and IDO. Concerning TNF-α, both TLR agonists induced a reduction of this pro-inflammatory cytokine, even if it did not reach statistical significance. Finally, only TLR4 triggering induced a significantly increased production of PGE_2_ compared with both hAMSCs alone or pre-treated with poly (I:C). These soluble factors were also evaluated as mRNA expression. Figure 2C depicts the fold change of mRNA expression by hAMSCs after triggering for 24 h with LPS or poly (I:C) compared with hAMSCs cultured with medium only. As shown, LPS induced significantly high mRNA expression of the anti-inflammatory cytokine IL-10 (10-fold increase) and of some chemokines, such as IP-10, and other factors such as IL-6 and COX-2. Poly (I:C) triggering induced the increase of all the factors analyzed; in particular, there was a high increase of IDO and IP-10 mRNA. Interestingly, after 48 h of LPS or poly (I:C) triggering, we observed a lower expression of all analyzed factors. In particular, a significant upregulation for COX-2 was observed after LPS treatment and for both IP10 and IL6 after poly (I:C) treatment. Moreover, differently to 24 h treatment, after 48 h of poly (I:C) treatment, we observed a significant downregulation of IDO confirming its protein expression (Figure 2D).

### *In vitro* Immunosuppressive Properties of hAMSCs Are Enhanced by Activated Immune Cells and by IFN-γ Priming

**Figure 3:**
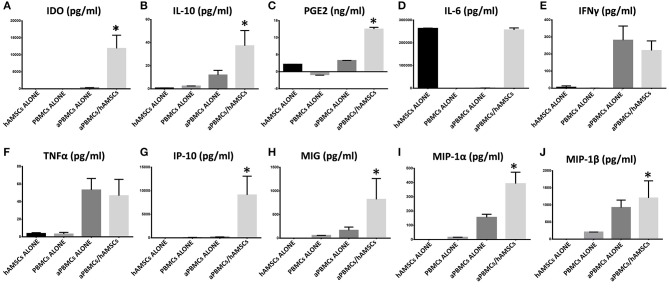
Expression analysis of immunomodulatory factors in PBMCs/hAMSCs co-coltures. Protein expressions were assayed in CM after 5 days of culture. **(A)** IDO expression (pg/ml). **(B)** IL-10 expression (pg/ml). **(C)** PGE_2_ expression (ng/ml). **(D)** IL-6 expression (pg/ml). **(E)** IFNγ expression (pg/ml). **(F)** TNFα expression (pg/ml). **(G)** IP-10 expression (pg/ml). **(H)** MIG expression (pg/ml). **(I)** MIP-1α expression (pg/ml). **(J)** MIP-1β expression (pg/ml). Human amnion mesenchymal stem cells grown alone (hAMSCs ALONE); peripheral blood mononuclear cells grown alone (PBMCs ALONE); peripheral blood mononuclear cells grown alone, activated with anti-CD3, anti-CD28, anti-CD40, and anti-BCR (aPBMCs ALONE); peripheral blood mononuclear cells activated with anti-CD3, anti-CD28, anti-CD40, and anti-BCR grown in co-cultures with human amnion mesenchymal stem cells (aPBMCs/hAMSCs). Data are means ± SD. ^*^p < 0.05 vs. aPBMCs/ALONE.

**Figure 4:**
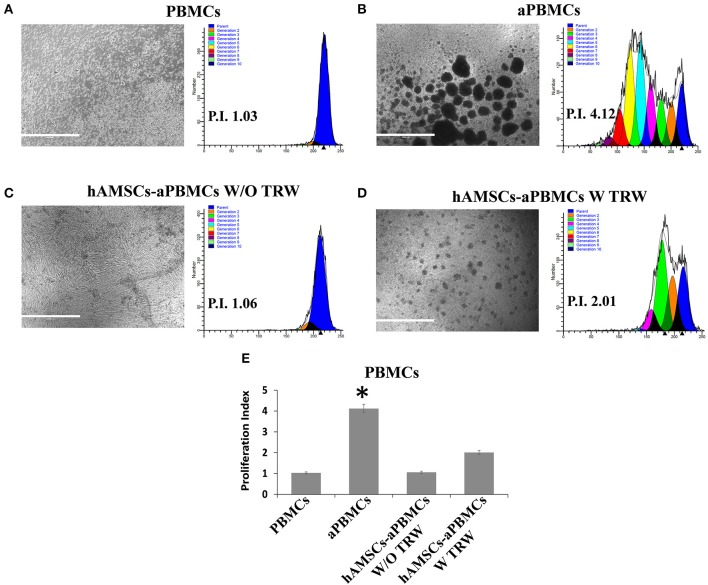
FACS analysis of PBMC proliferation co-cultured with hAMSCs. **(A)** Representative images and respective FACS graphic of unstimulated PBMCs grown alone (PBMCs). **(B)** Representative images and respective FACS graphic of PBMCs grown alone and activated with anti-CD3, anti-CD28, anti-CD40, and anti-BCR (aPBMCs). **(C)** Representative images and respective FACS graphic of hAMSCs co-cultured with PBMCs activated with anti-CD3, anti-CD28, anti-CD40, and anti-BCR, without Transwell (hAMSCs-aPBMCs W/O TRW). **(D)** Representative images and respective FACS graphic of hAMSCs co-cultured with PBMCs activated with anti-CD3, anti-CD28, anti-CD40, and anti-BCR, with Transwell (hAMSCs-aPBMCs W TRW). **(E)** Proliferation index of PBMCs. Data are means ± SD. ^*^p < 0.05 vs. unstimulated PBMCs. P.I., proliferation index; bar = 1,000 μm.

**Figure 5:**
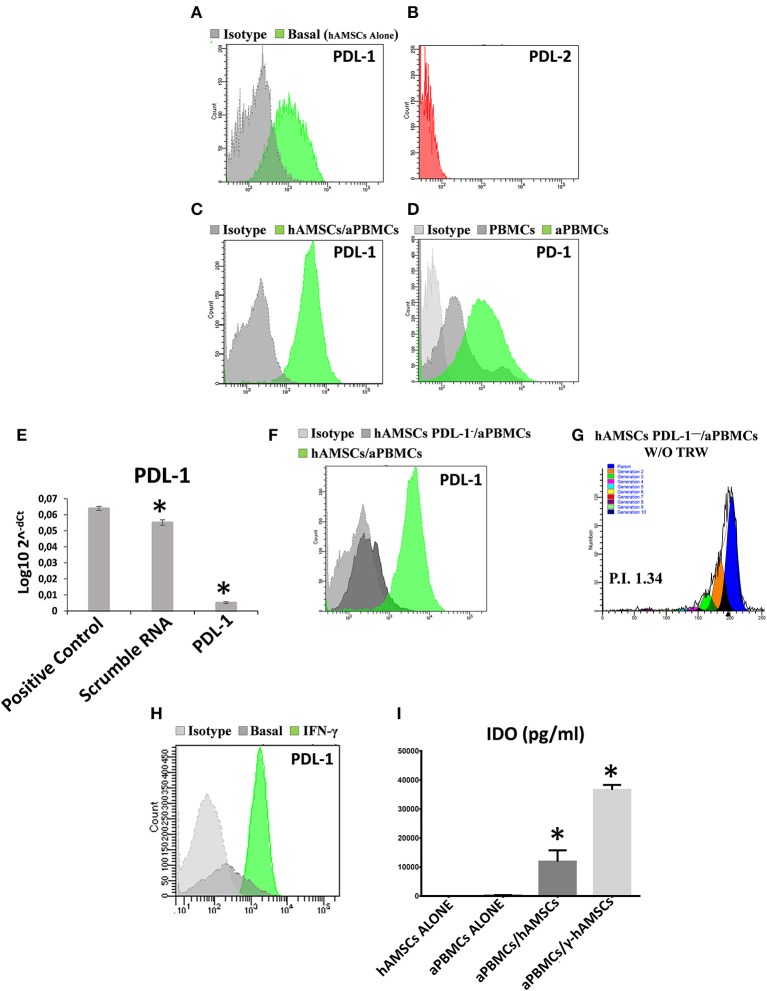
Analysis of both PDL-1 and PD-1 expression in hAMSCs and PBMCs, analysis of PDL-1 in hAMSCs PDL-1^−^ and IDO production analysis. **(A)** Representative FACS graphic of PDL-1 expression in hAMSCs grown alone (hAMSCs alone). **(B)** Representative FACS graphic of PDL-2 expression in hAMSCs grown alone. **(C)** Representative FACS graphic of PDL-1 expression in hAMSCs grown in co-cultures with PBMCs activated with anti-CD3, anti-CD28, anti-CD40, and anti-BCR (hAMSCs/aPBMCs). **(D)** Representative FACS graphic of PD-1 expression in PBMCs grown alone. **(E)** Expression of PDL-1 in hAMSCs after PDL-1 silencing. **(F)** Representative FACS graphic of PDL-1 expression in hAMSCs with or without CD274 silencing and in co-cultures with aPBMCs. **(G)** FACS graphic of aPBMCs proliferation co-cultured with hAMSCs PDL-1^−^ without Transwell. **(H)** Representative FACS graphic of PDL-1 expression in hAMSCs activated with IFNγ. **(I)** IDO expression (pg/ml) in aPBMCs co-cultured with hAMSCs activated with IFNγ (γ-hAMSCs). Data are means ± SD. ^*^p < 0.05 vs. positive control **(E)** or hAMSCs alone **(I)**. P.I., proliferation index.

The immunomodulatory ability of hAMSCs was evaluated in co-culture using PBMCs from different healthy donors. PBMCs labeled with CFSE were activated with anti-CD3, anti-CD28, anti-BCR, and anti-CD40 monoclonal antibodies, and incubated for 5 days on a layer of hAMSCs. To shed light on the contribution of paracrine factors to immunosuppression, the supernatants were collected. As shown in Figure 3, the activated PBMCs enhanced hAMSC immunomodulatory properties, as there were significantly high increases of the main paracrine factors involved in immunomodulation, such as IDO (A), IL-10 (B), and PGE_2_ (C), which, instead, are produced at lower levels in both hAMSCs alone and activated PBMCs alone. Concerning IL-6 (D), hAMSCs are the sole cells that contributed to its production, with no modification when cells were in co-culture. On the contrary, activated PBMCs alone contributed to the production of two important pro-inflammatory cytokines: IFN-γ (E) and TNF-α (F). In co-culture, the levels of these two cytokines slightly, but not significantly, decreased, due likely to a negative feedback induced by the hAMSC immunomodulatory enabling. Moreover, hAMSC/activated PBMC co-culture also induced the production, at high and significant levels, of some chemokines, such as IP-10, MIG, MIP-1α, and MIP-1β (Figures 3G–J), involved in the chemotaxis of innate immune cells. These data were confirmed by analysis of PBMC proliferation. After 5 days of co-culture with hAMSCs, as shown in Figure 4, there was a total inhibition of PBMC proliferation, as evidenced by the proliferation index (P.I. = 1.06) (C), comparable to that of negative control (A). To assess whether the inhibitory effect of hAMSCs was mediated only by soluble factors or by cell-to-cell contact, we used a Transwell system in which hAMSCs were co-cultured with activated PBMCs placed in Transwell inserts. As shown in Figure 4D, PBMC proliferation was only partially inhibited (P.I. = 2.01; 50% of PBMC alone activated, P.I. = 4.12). These data suggest that the hAMSCs, in order to exert their complete immunomodulatory effect, need direct contact and soluble factor, as well. In this regard, we found that hAMSCs constitutively express PDL-1 (Figure 5A), but not PDL-2 (Figure 5B). Moreover, when in co-culture with activated PBMCs, hAMSCs showed increased expression of PDL-1 (Figure 5C). Concerning the expression of PD-1, this molecule is expressed at low levels in resting PBMCs and, as expected, PD-1 expression increased after activation (Figure 5D). In order to demonstrate the involvement of PDL-1 in the cell-to-cell inhibition of PBMC proliferation, we silenced the PDL-1 gene in hAMSCs. After evaluating the gene silencing by RT-PCR (Figure 5E) and the consequent reduced expression of PDL-1 molecule in hAMSC membrane (Figure 5F), we performed co-culture of activated PBMCs with hAMSCs silenced for PDL-1 gene. As depicted in Figure 5G, the proliferative ability of PBMC is partially conserved (P.I. = 1.34), differently from non-Transwell co-culture (Figure 4C), demonstrating the important role of the PDL-1/PD-1 axis in the immunosuppressive ability of hAMSCs.

Finally, after the demonstration that IFN-γ is the main cytokine produced by activated PBMCs in co-culture, we wanted to confirm its effect on the enhancement of hAMSC immunomodulatory functions. We placed in co-culture PBMCs IFN-γ primed hAMSCs (γ-hAMSCs), and after 48 h of IFN-γ priming, hAMSCs showed an increased expression of PDL-1 (Figure 5H), and a significantly high increase of IDO (Figure 5I). Moreover, we found that IFN-γ treatment was capable of inducing the upregulation of different EXO-derived miRNAs involved in immunomodulation.

### hAMSCs Induce a Remodeling of Immune System Cells Toward an Anti-inflammatory Phenotype

**Figure 6:**
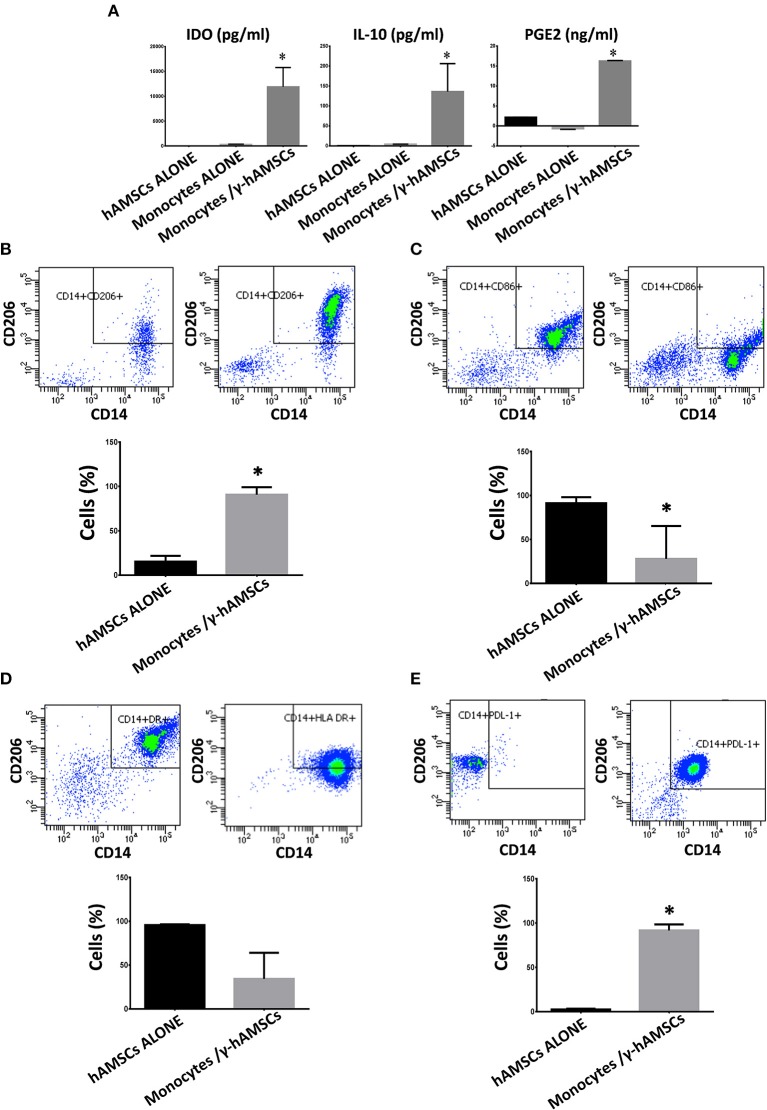
Expression analysis of immunomodulatory factors and phenotyping of monocytes co-cultured with hAMSCs activated with IFNγ (γ-hAMSCs). Protein expression was assayed in CM after 5 days of culture. **(A)** IDO, IL-10, and PGE_2_ expression in CM derived from monocytes/γ-hAMSC co-cultures. **(B)** FACS analysis of CD14^+^/CD206^+^ monocytes grown alone (left panel) or in co-cultures with γ-hAMSCs (right panel). **(C)** FACS analysis of CD14^+^/CD86^+^ monocytes grown alone (left panel) or in co-cultures with γ-hAMSCs (right panel). **(D)** FACS analysis of CD14^+^/HLA DR^+^ monocytes grown alone (left panel) or in co-cultures with γ-hAMSCs (right panel). **(E)** FACS analysis of CD14^+^/PDL-1^+^ monocytes grown alone (left panel) or in co-cultures with γ-hAMSCs (right panel). Data are means ± SD.

As discussed above, in hAMSC/activated PBMC co-culture, there is a significantly high production of the main paracrine factors involved in immunomodulation (IL-10, PGE_2_, and IDO). Since hAMSCs alone do not meaningfully produce these three molecules, we investigated the origin of IL-10, PGE_2_, and IDO detected at high concentration. Many studies have found that MSCs can influence other cells to produce immunomodulatory factors. As among PBMCs, other than lymphoid cells, there are also myeloid cells, such as monocytes. In order to determine the source of soluble immunomodulatory molecules, we performed co-culture of γ-hAMSCs with purified CD14^+^ monocytes. As depicted in Figure 6A, the interaction between γ-hAMSCs and monocytes seems to be responsible for the production of the immunomodulatory molecules IDO, PGE2, and, in particular, IL-10, which were highly increased compared with those found in hAMSC/total activated PBMCs co-culture (100 vs. 50 pg/ml). The effect of the interplay between hAMSCs and monocytes is reflected also in the phenotype and functional remodeling of these latter cells. As shown in Figure 6B, there was a high increase of CD14^+^/CD206^+^ M2-like anti-inflammatory monocytes, which are widely known to be IL-10 high producer cells (36). Moreover, on CD14^+^ monocytes, pre-activated with γ-hAMSCs, we found a significantly reduced expression of the co-stimulatory molecule CD86 (Figure 6C), a reduction, though not significant, of HLA-DR surface expression (Figure 6D), and a significant increase of the immunomodulatory molecule PDL-1 (Figure 6E).

**Figure 7:**
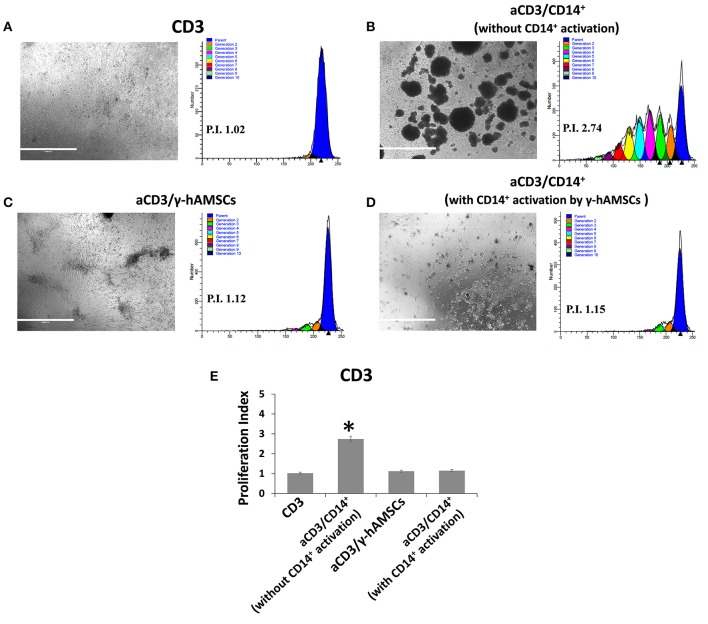
FACS analysis of CD3 cells proliferation co-cultured with CD14^+^ monocytes grown with or without γ-hAMSCs. **(A)** Representative images and respective FACS graphic of unstimulated CD3 cells grown alone (CD3). **(B)** Representative images and respective FACS graphic of CD3 cells stimulated with anti-CD3, anti-CD28, anti-CD40, and anti-BCR (aCD3) co-cultured with monocytes CD14^+^. **(C)** Representative images and respective FACS graphic of aCD3 co-cultured with hAMSCs activated with IFNγ (γ-hAMSCs). **(D)** Representative images and respective FACS graphic of aCD3 co-cultured with monocytes CD14^+^ pre-stimulated by co-cultures with γ-hAMSCs. **(E)** Proliferation index of CD3. Data are means ± SD. ^*^p < 0.05 vs. unstimulated CD3. P.I., proliferation index; bar = 1,000 μm.

To test whether CD14^+^/CD206^+^ monocytes, induced by activated hAMSCs, themselves have immunomodulatory activity, we first co-cultured resting CD14^+^ monocytes with γ-hAMSC. After 5 days of co-culture, only monocytes were harvested and re-plated for another 5 days with activated CFSE-labeled CD3^+^ T lymphocytes. As shown in Figure 7, CD14^+^ monocytes derived from γ-hAMSCs co-culture (CD14^+^/CD206^+^ M2-like monocytes) acquired the ability to inhibit CD3^+^ T cell proliferation.

### Exosomes Derived From IFN-γ-Primed hAMSCs Contain miRNAs Potentially Involved in Immunomodulation

**Figure 8:**
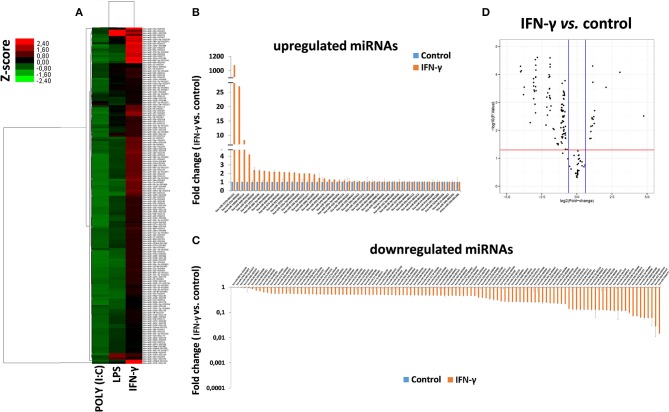
Differentially expressed miRNAs in EXO derived from hAMSCs. **(A)** Hierarchical clustering based on *Z* ratios (calculated as described in the *Materials and Methods* section) of miRNA expression levels in EXO derived from hAMSCs treated with poly (I:C), LPS, and IFNγ. **(B)** Upregulated miRNAs in EXO derived from hAMSCs treated with IFNγ. **(C)** Downregulated miRNAs in EXO derived from hAMSCs treated with IFNγ. **(D)** Volcano plot analysis of deregulated miRNAs in EXO derived from hAMSCs treated with IFNγ (*p* ≤ 0.05 and fold change ≥ 1.5). miRNA levels were normalized to those of U6, and expressed as fold change vs. untreated cells (control). Data are means ± SD.

To study the miRNA expression profile of EXO derived from γ-hAMSCs, we partially analyzed their miRNome with TaqMan Low Density Arrays. Statistical analyses were done on 160 miRNAs selected from 754 miRNAs after quality control screening (amplification score > 1.1, Cq confidence > 0.8). Hierarchical clustering analysis showed systematic variations in the miRNA expression among poly (I:C), LPS and IFN-γ treatment, with the latter being very different from the others (Figure 8A). We identified 46 upregulated miRNAs (Figure 8B) and 114 downregulated miRNAs (Figure 8C) in γ-hAMSCs. Then, volcano plot analysis (*p* < 0.05 and fold change > 1.5) identified 17 miRNAs upregulated in γ-hAMSCs (Figure 8D), and on these, the subsequent analyses were directed. In particular, we performed GO analysis using the online software mirPath (v.3) from DIANA tools (http://diana.imis.athena-innovation.gr) (37). The 17 upregulated miRNAs were enriched in the BP, CC, and MF terms associated with immune system processes, with a *p* value threshold of 0.05. These miRNAs were highly associated with immune and inflammatory response/regulation pathways. The top 10 GO terms involved in immune system regulation obtained by GO enrichment analysis are shown in Table 1.

**Table 1:** Partial list of GO enrichment analysis (GO category analyzed by mirPath of DIANA TOOLS) for the deregulated genes.

**GO category**	***p* value**	**Genes**	**miRNAs**	**miRNA names**
Immune system process (GO:0002376)	2.30E−15	537	16	hsa-miR-125a-5p|Tarbase;hsa-miR-125b-5p|Tarbase; hsa-miR-130b-3p|Tarbase;hsa-miR-133a-3p|TargetScan; hsa-miR-140-3p|Tarbase; hsa-miR-200c-5p|Tarbase; hsa-miR-223-3p|Tarbase; hsa-miR-224-5p|Tarbase; hsa-miR-23a-3p|Tarbasehsa-miR-26b-5p|Tarbase; hsa-miR-320a|Tarbase;hsa-miR-517b-3p|TargetScan; hsa-miR-574-3p|Tarbase; hsa-miR-668-3p|TargetScan;hsa-miR-6886-3p|TargetScan; hsa-miR-99a-5p|Tarbase
Innate immune response (GO:0045087)	4.29E−5	226	15	hsa-miR-125a-5p|Tarbase; hsa-miR-125b-5p|Tarbase; hsa-miR-130b-3p|Tarbase; hsa-miR-133a-3p|TargetScan; hsa-miR-140-3p|Tarbase; hsa-miR-200c-5p|Tarbase; hsa-miR-223-3p|Tarbase; hsa-miR-224-5p|Tarbase; hsa-miR-23a-3p|Tarbase; hsa-miR-26b-5p|Tarbase; hsa-miR-320a|Tarbase; hsa-miR-574-3p|Tarbase; hsa-miR-668-3p|TargetScan; hsa-miR-6886-3p|TargetScan; hsa-miR-99a-5p|Tarbase
Cytokine-mediated signaling pathway (GO:0019221)	2.77E−5	109	15	hsa-miR-125a-5p|Tarbase; hsa-miR-125b-5p|Tarbase; hsa-miR-130b-3p|Tarbase; hsa-miR-133a-3p|TargetScan; hsa-miR-140-3p|Tarbase; hsa-miR-200c-5p|Tarbase; hsa-miR-223-3p|Tarbase; hsa-miR-224-5p|Tarbase; hsa-miR-23a-3p|Tarbase; hsa-miR-26b-5p|Tarbase; hsa-miR-320a|Tarbase; hsa-miR-517b-3p|TargetScan; hsa-miR-574-3p|Tarbase; hsa-miR-6886-3p|TargetScan; hsa-miR-99a-5p|Tarbase
Toll-like receptor signaling pathway (GO:0002224)	6.30E−8	48	13	hsa-miR-125a-5p|Tarbase; hsa-miR-125b-5p|Tarbase; hsa-miR-130b-3p|Tarbase; hsa-miR-140-3p|Tarbase; hsa-miR-200c-5p|Tarbase; hsa-miR-223-3p|Tarbase; hsa-miR-224-5p|Tarbase; hsa-miR-23a-3p|Tarbase; hsa-miR-26b-5p|Tarbase; hsa-miR-320a|Tarbase; hsa-miR-574-3p|Tarbase; hsa-miR-6886-3p|TargetScan; hsa-miR-99a-5p|Tarbase
Leukocyte migration (GO:0050900)	3.00E−6	47	12	hsa-miR-125a-5p|Tarbase; hsa-miR-125b-5p|Tarbase; hsa-miR-130b-3p|Tarbase; hsa-miR-140-3p|Tarbase; hsa-miR-223-3p|Tarbase; hsa-miR-224-5p|Tarbase; hsa-miR-23a-3p|Tarbase; hsa-miR-26b-5p|Tarbase; hsa-miR-320a|Tarbase; hsa-miR-574-3p|Tarbase; hsa-miR-668-3p|TargetScan; hsa-miR-99a-5p|Tarbase
Antigen processing and presentation of exogenous peptide antigen via MHC class II (GO:0019886)	5.93E−6	50	12	hsa-miR-125a-5p|Tarbase; hsa-miR-125b-5p|Tarbase; hsa-miR-130b-3p|Tarbase; hsa-miR-133a-3p|TargetScan; hsa-miR-140-3p|Tarbase; hsa-miR-200c-5p|Tarbase; hsa-miR-223-3p|Tarbase; hsa-miR-224-5p|Tarbasehsa-miR-23a-3p|Tarbase; hsa-miR-26b-5p|Tarbase; hsa-miR-320a|Tarbase; hsa-miR-574-3p|Tarbase
MyD88-dependent toll-like receptor signaling pathway (GO:0002756)	1.31E−6	38	12	hsa-miR-125a-5p|Tarbase; hsa-miR-125b-5p|Tarbase; hsa-miR-130b-3p|Tarbase; hsa-miR-140-3p|Tarbase; hsa-miR-200c-5p|Tarbase; hsa-miR-224-5p|Tarbase; hsa-miR-23a-3p|Tarbase; hsa-miR-26b-5p|Tarbase; hsa-miR-320a|Tarbase; hsa-miR-517b-3p|TargetScan; hsa-miR-6886-3p|TargetScan; hsa-miR-99a-5p|Tarbase
Positive regulation of type I interferon production (GO:0032481)	5.5E−3	23	12	hsa-miR-125a-5p|Tarbase; hsa-miR-125b-5p|Tarbase; hsa-miR-130b-3p|Tarbase; hsa-miR-133a-3p|TargetScan; hsa-miR-140-3p|Tarbase; hsa-miR-200c-5p|Tarbase; hsa-miR-23a-3p|Tarbase; hsa-miR-26b-5p|Tarbase; hsa-miR-320a|Tarbase; hsa-miR-668-3p|TargetScan; hsa-miR-6886-3p|TargetScan; hsa-miR-99a-5p|Tarbase
IFN-γ-mediated signaling pathway (GO:0060333)	1.3E−3	25	10	hsa-miR-125a-5p|Tarbase; hsa-miR-125b-5p|Tarbase; hsa-miR-130b-3p|Tarbase; hsa-miR-140-3p|Tarbase; hsa-miR-224-5p|Tarbase; hsa-miR-23a-3p|Tarbase; hsa-miR-26b-5p|Tarbase; hsa-miR-320a|Tarbase; hsa-miR-6886-3p|TargetScan; hsa-miR-99a-5p|Tarbase
JAK-STAT cascade involved in growth hormone signaling pathway (GO:0060397)	4.3E−3	11	9	hsa-miR-125a-5p|Tarbase; hsa-miR-125b-5p|Tarbase; hsa-miR-130b-3p|Tarbase; hsa-miR-140-3p|Tarbase; hsa-miR-223-3p|Tarbase; hsa-miR-23a-3p|Tarbase; hsa-miR-26b-5p|Tarbase; hsa-miR-320a|Tarbase; hsa-miR-6886-3p|TargetScan

**Figure 9:**
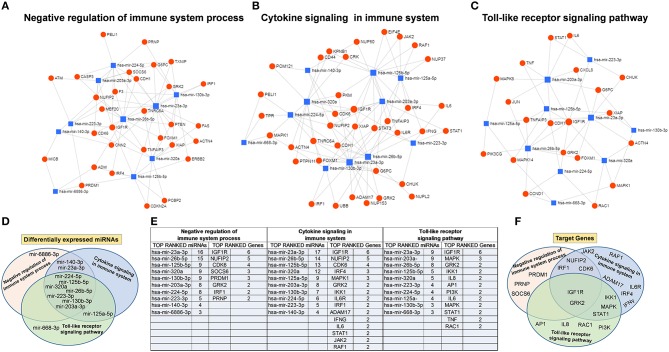
Protein–protein interaction network generated for shared miRNA target genes after miRNET analysis. Only genes targeted by at least two differentially expressed miRNAs are shown. Upper images show networks with all interactions between deregulated miRNAs and genes involved in **(A)** negative regulation of immune system process, **(B)** cytokine signaling in immune system, and **(C)** toll-like receptor signaling pathway. **(D)** Distribution of the top-ranked deregulated miRNAs in the three analyzed pathways. **(E)** Table summarizing both the top-ranked miRNAs and the top-ranked genes showed in the network analysis. **(F)** Distribution of the top-ranked target genes in the three analyzed pathways.

**Figure 10:**
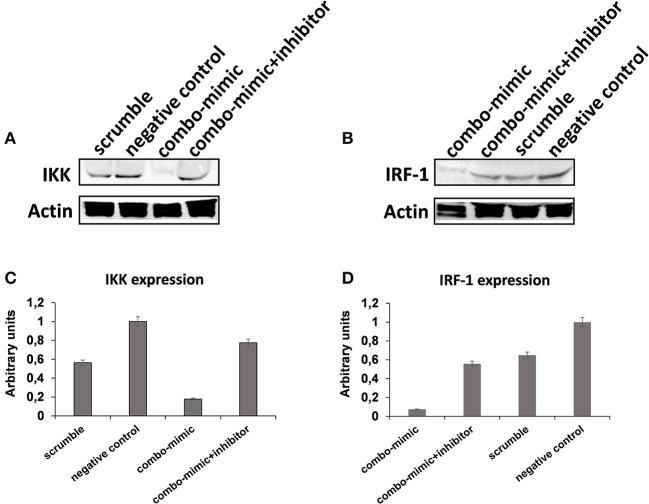
Western blot analysis of IKK, IRF-1, and Actin protein in whole lysates of purified monocytes. **(A)** Western blot image of IKK and Actin protein. **(B)** Western blot image of IRF-1 and Actin protein. **(C)** IKK Western blot results quantified by ImageJ software. **(D)** IRF-1 Western blot results quantified by ImageJ software. Protein expression was normalized to β-Actin, and the ratio to relevant control was presented as fold changes **(C,D)**. In the combo-mimic treatment for IKK, the cells were transfected with miR-223-3p and miR-23a-3p. In the combo-mimic treatment for IRF-1, the cells were transfected with miR-23a-3p, miR-130b-3p, and miR-125b-5p.

**Figure 11:**
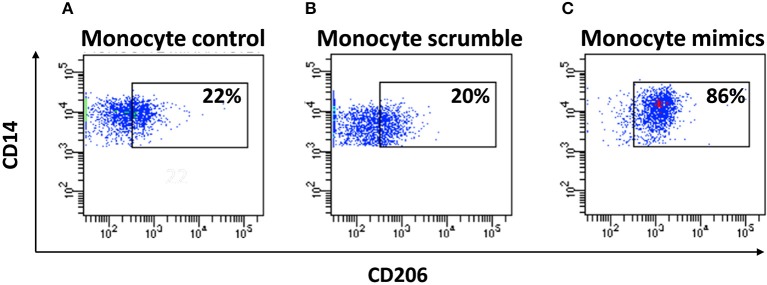
FACS analysis of purified miRNA-transfected monocytes. **(A)** FACS analysis of CD14^+^/CD206^+^ untransfected monocytes. **(B)** FACS analysis of CD14^+^/CD206^+^ monocytes transfected with scrumble. **(C)** FACS analysis of CD14^+^/CD206^+^ monocytes transfected with miR-130b-3p, miR-26b-5p, miR-125b-5p, miR-203a-3p, miR-23a-3p, and miR-223-3p.

To understand the potential involvement of the differentially expressed miRNAs in the regulation of the immune system, we also investigated, using the miRNET database, a miRNA–target network of representative pathways involved in immune system regulation: (1) negative regulation of immune system process; (2) cytokine signaling in the immune system; and (3) toll-like receptor signaling pathway (Figures 9A–C). Only seven miRNAs were correlated with all three analyzed pathways (Figure 9D). Furthermore, we identified potential targets for upregulated miRNAs. One mutual gene, IGF1R, is common to all three analyzed pathways and was predicted to be targeted by six miRNAs, while GRK2 is also common to all three studied pathways, but it was targeted by two miRNAs. NUFIP2, CDK6, and IRF1 are common to two analyzed pathways and targeted by five, four, and two miRNAs, respectively, as well as MAPK, IKK1, and STAT1, which are targeted by three and two miRNAs, respectively. All other genes (PRDM1, PRNP, SOCS6, RAF1, IL6R, JAK2, ADAM17, IRF4, IFN-γ, AP1, IL8, RAC1, and PI3K) were lacking specific pathways and targeted by at least two miRNAs (Figures 9E,F). Moreover, we investigate the effects of these deregulated miRNA on monocyte behavior. We found that the transfection of purified monocytes with specific miRNA is able to downregulate the expression of two key proteins involved in monocyte polarization. Indeed, as shown in Figure 10, using a combination of miR-223-3p and miR-23a-3p, we observed a downregulation of IKK protein (Figures 10A,C), and using another combination of miRNAs (miR-23a-3p, miR-130b-3p, and miR-125b-5p), we observed a downregulation of IRF-1 protein (Figures 10B,D). To demonstrate the potential effects of the six upregulated γ-hAMSC-derived miRNA on monocyte polarization, we performed a combined transfection with miR-130b-3p, miR-26b-5p, miR-125b-5p, miR-203a-3p, miR-23a-3p, and miR-223-3p on purified monocytes. As shown in Figure 11, we observed a high increase of CD14^+^/CD206^+^ M2-like monocytes (Figure 11C) on transfected cells compared to both untransfected (Figure 11A) and scrumble transfected (Figure 11B) cells.

## Discussion

Human mesenchymal stromal cells can be isolated from a variety of adult tissues and currently represent the prototype cells in regenerative medicine, being the most widely studied cell type in both preclinical and clinical trials (38, 39). Placenta is a source of MSCs that offer many advantages with respect to other adult MSCs. The isolation of placenta-derived MSCs does not involve the use of invasive methods, and the isolated cells are a homogeneous population with a higher proliferative rate in culture compared with MSCs from other tissues (24, 40). This feature makes it possible to obtain a great number of cells in fewer passages, reducing the risk of *ex vivo* senescence (41). In addition, placenta-derived MSCs are younger cells, and have not been exposed to harmful agents and other stressors, resulting in a higher clinical efficacy and safety compared with MSCs from other tissues (42).

sPlacenta-derived MSCs, as well as other MSCs, exhibit the particular ability to produce potent immunosuppressive effects. When MSCs are co-cultured with stimulated PBMCs, they inhibit the immune cells' proliferative response both directly and indirectly (43–45). It is known that MSCs directly affect immune cells' response by expressing a plethora of soluble immunosuppressive factors such as IDO, which is not constitutive expressed by MSCs, but depends on exposure to IFN-γ (46); prostaglandin E_2_ (PGE_2_), a metabolite of arachidonic acid generated through a cyclo-oxygenase-2 (COX-2)-regulated enzymatic cascade (47); and IL-10 (48). IDO and PGE_2_ are involved in metabolic reprogramming and are directly produced by MSCs (48). The production of the anti-inflammatory cytokine IL-10 depends on the experimental inflammatory conditions that MSCs are exposed to, and its role in the immunosuppressive activity can be indirect, resulting from immune cells in co-culture with MSCs (48, 49). Beyond direct production of soluble proteins, MSCs also release EXO, which produce different effects in a paracrine manner by influencing inflammatory processes. It has been shown that EXO, from specific cell types and conditions, have positive effects on regeneration of many tissues (50). Bian et al. showed that bone marrow MSC-derived EXO regulate inflammation and promote angiogenesis in a rat myocardial infarction model (51). Moreover, MSCs can exert their immunomodulatory ability by direct cell-to-cell interaction through different molecular receptors, such as programmed death-ligand 1 (PDL-1) (52). Finally, MSCs have an important role in the recruitment and activation of other immunoregulatory cells (e.g., myeloid cells) that, in turn, can enhance the immunosuppression and sustain this activity in a longer term, explaining the therapeutic benefit even beyond the persistence of MSCs (48). Therefore, the key to the understanding of MSC efficacy for tissue repair or in the cure/prevention of inflammatory disease is to discriminate how MSCs modulate the inflammatory niche. The effects of MSCs on both innate and specific immune responses are suggested by their direct interaction with almost all the cells of the immune system, such as T and B lymphocytes, natural killer cells, dendritic cells, monocytes/macrophages, and neutrophils (53). It is unclear whether MSCs should be categorized as immunosuppressive, or if they induce an immune tolerance, implying a more specific suppression of aberrant immune responses. Furthermore, there is a need to explain whether the immunomodulatory ability of MSCs is dependent on a number of factors, including MSC activation, MSC tissue of origin, and MSC contact with cells of the immune system.

In our study, we aimed to establish the potential role of a particular population of placenta-derived MSCs, namely, hAMSCs in immunomodulation. We found that the immunosuppressive properties of hAMSCs are not constitutive, but require supportive signals capable of promoting these properties. Indeed, we show that hAMSCs alone are not able to produce a proper amount of soluble factors involved in immunomodulation, such as IL-10, IDO, or PGE_2_, but only produce high levels of IL-6. This is a well-known pro-inflammatory cytokine, though, in some cases, IL-6 can also exert an indirect suppressive function, reducing the stimulating effects of dendritic cells on T lymphocytes by inducing anergy in the latter cells (1). The nature of the supportive signals is strictly related to inflammation and immune response. This is why the surrounding environment is indispensable for hAMSC immunosuppressive licensing (20, 46).

Among the mechanisms involved in the response to inflammatory stimuli, TLR triggering may play an important role (14, 15). We found that the stimulation of both TLR3 and TLR4 on hAMSCs induces the transcription of numerous genes involved in immunomodulation, but this stimulation is not enough in terms of paracrine factors (cytokines, EXO-derived miRNAs) production. The strong immunomodulatory licensing signal comes from the direct interaction between hAMSCs and stimulated PBMCs. The inflammatory environment, due to the pro-inflammatory cytokines produced by activated immune cells, seems to play a pivotal role in hAMSC immunosuppressive properties. In the presence of activated PBMCs, hAMSCs start to synthesize the immunosuppressive soluble factors IDO, PGE_2_, and IL-10, which are unable to produce at a steady state. We found that the immunomodulatory effect of hAMSCs does not depend solely on the secretion of soluble factors. In fact, using a Transwell insert (to physically separate hAMSCs from PBMCs), we found that PBMC proliferation is not completely inhibited, suggesting an important role played also by cell-to-cell contact. In this regard, we observed both the upregulation of PDL-1 on hAMSCs and of PD-1 on activated PBMCs when these cells were in co-culture, as previously reported (54, 55). Moreover, our data show that the knockdown of PDL-1 gene, using a specific siRNA, induces a significantly reduced expression of PDL-1 on hAMSCs, a condition that is mirrored by the results obtained in hAMSC/activated PBMC co-culture. In particular, we show how PBMCs' proliferative ability is partially conserved, demonstrating the important contribution of the PDL-1/PD-1 axis in the immunomodulatory effects of “licensed” hAMSCs. These results suggest that the immunomodulatory effect of hAMSCs seems to be the consequence of a synergic effect mediated by both soluble factors and direct cell contact.

**Figure 12:**
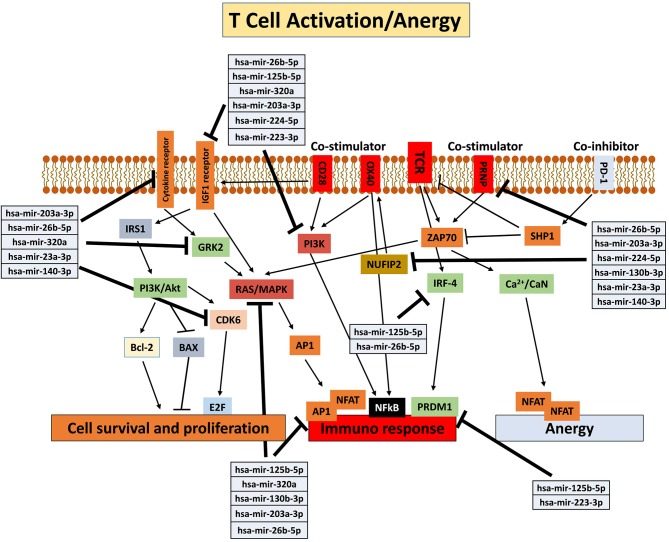
Schematic representation of T cell activation/anergy pathways modified from KEGG PATHWAY Database (https://www.genome.jp/kegg/pathway.html). Deregulated miRNAs and corresponding genes target are shown.

**Figure 13:**
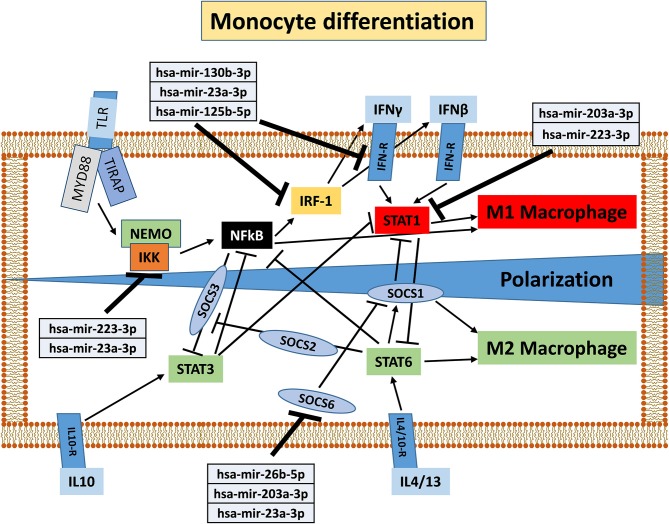
Schematic representation of monocyte differentiation toward M1 or M2 phenotype modified from KEGG PATHWAY Database (https://www.genome.jp/kegg/pathway.html). Deregulated miRNAs and corresponding target genes are shown.

The effect of the pro-inflammatory environment on hAMSC immunomodulation and the well-established role of IFN-γ in the enhancement of MSC suppressive activities (1, 19, 46, 48, 55) prompted us to directly pre-activate hAMSCs with IFN-γ in order to verify whether these cells improve their immunomodulatory activity. The IFN-γ priming of hAMSCs (γ-hAMSCs) induces the increase of PDL-1 expression, a significantly high production of IDO, and the upregulation of different EXO-derived miRNAs involved in immunomodulation. In order to examine the potential involvement of γ-MSC-derived EXO in the hAMSC immunomodulatory paracrine effects, we analyzed the expression of EXO-deregulated miRNAs in relation to their interaction with immunoregulatory pathways. Our miRNA–target network analysis revealed that among the 17 deregulated miRNAs, 9 miRNAs are involved in the regulation of key proteins that control both the T cell activation/anergy pathway and the monocyte differentiation pathway. In particular, 9 miRNAs interact with IGF1R, PI3K, GRK2, CDK6, RAS/MAPK, AP1, PRDM1, NUFIP2, PRNP, and IRF-4, genes that govern T cell survival/proliferation, T cell immune response, and T cell anergy (56–59) (Figure 12). Furthermore, we also found that, among these upregulated miRNAs, six of them interact with key genes involved in a crucial immune tolerance process: monocyte differentiation that produces inflammatory M1 or regulatory M2 cells. We investigated this hypothesis transfecting purified monocytes with these six deregulated miRNAs. Our data showed not only that the transfection with miR-223-3p, miR-23a-3p, miR-130b-3p, and miR-125b-5p was able to downregulate both IKK and IRF-1 protein (key regulators of monocytes polarization), but we also demonstrated that the transfection with a combination of miR-130b-3p, miR-26b-5p, miR-125b-5p, miR-203a-3p, miR-23a-3p, and miR-223-3p was able to induce an M2-like phenotype. Interestingly, this effect was not observed when monocytes were transfected with singular miRNA or when these cells were transfected with partial combination of the above-described miRNAs (data not shown). In Figure 13, we depict a schematic representation of the six deregulated miRNAs that interact with IRF-1, IFN-R, IKK, STAT1, and SOCS6, genes that govern monocyte differentiation toward M1 or M2 phenotype (60). Overall, these data show a potential intervention of EXO-derived miRNAs on the paracrine immunomodulatory effects of hAMSCs.

The interaction of hAMSCs with the pro-inflammatory environment, and the subsequent immunoregulatory behavior of these cells, clearly leads us to consider that there is a mutual feedback mechanism between hAMSCs and the immune system cells. For this reason, it cannot be assumed that hAMSCs exert only a direct inhibitory effect on effector cells of immune response, but, due to the soluble factor that hAMSCs produce, these cells have an important impact on the remodeling and recruitment of other cells involved in immune regulation, which, in turn, can enhance and sustain immunoregulatory activity for a longer time. In this study, we demonstrate how monocytes play a critical role for the delivery of hAMSC immunomodulatory activity. Interestingly, we found a highly significant production of some chemokines involved in the innate immunity cell chemotaxis (IP-10, MIG, MIP-1α, and MIP-1β) in hAMSC/activated PBMC co-culture. Moreover, we found that IFN-γ-activated hAMSCs produce high levels of IDO, which has been found to induce anti-inflammatory monocytes (36). Furthermore, it has been shown that MSC-derived EXO induce an M2-like phenotype in monocytes *in vitro*, resulting in polarization of activated CD4 T cells to regulatory T cells (61). Based on these data, we co-cultured γ-hAMSCs with purified CD14^+^ monocytes. The effect of this direct interaction is responsible for the increased production of the anti-inflammatory cytokine, IL-10, and the phenotypic and functional modification of monocytes. In fact, we found a high increase of CD14^+^/CD206^+^ M2-like anti-inflammatory monocytes, which simultaneously downregulate the expression of the co-stimulatory molecule CD86 and upregulate the immunosuppressive molecule PDL-1. Moreover, these CD14^+^/CD206^+^ monocytes, deriving from a co-culture with γ-hAMSCs, when harvested and re-plated with activated CD3^+^ T lymphocytes, acquire the ability to themselves induce immunomodulatory activity. Indeed, we demonstrate how γ-hAMSCs-derived M2-like monocytes are able to inhibit CD3^+^ T cell proliferation.

The extensive implications of MSCs in modulating different aspects of the immune system make these cells attractive candidates to be employed in therapeutic application in immune-based diseases. The clinical applications of MSCs have highlighted some controversial results. *In vivo* effects of MSCs depend on the context of the disease microenvironment, as described in different clinical trials (62, 63). Our study leads us to hypothesize that hAMSCs could become a useful tool for treating different inflammatory diseases, including end-stage pathologies or adverse effects in transplanted patients. Further studies are necessary to explore whether it is better to use IFN-γ primed hAMSCs products, such as γ-hAMSCs-derived EXO or autologous M2-like monocytes pre-treated with γ-hAMSCs, than a direct hAMSCs infusion.

## Data Availability Statement

The raw data supporting the conclusions of this article will be made available by the authors, without undue reservation, to any qualified researcher.

## Author Contributions

MB and VM conceived and designed experiments, analyzed and interpreted data, and drafted the article. MP collected and processed placenta samples. GA performed silencing experiments, analyzed and interpreted data, and drafted the article. MB, VM, AG, GA, and CC performed cellular and molecular experiments. PC revised the paper critically for important intellectual content. All authors have seen and approved the final draft of the manuscript.

### Conflict of Interest

The authors declare that the research was conducted in the absence of any commercial or financial relationships that could be construed as a potential conflict of interest.
